# Degradation of Aflatoxin B_1_ and Zearalenone by Bacterial and Fungal Laccases in Presence of Structurally Defined Chemicals and Complex Natural Mediators

**DOI:** 10.3390/toxins11100609

**Published:** 2019-10-22

**Authors:** Xiaolu Wang, Yingguo Bai, Huoqing Huang, Tao Tu, Yuan Wang, Yaru Wang, Huiying Luo, Bin Yao, Xiaoyun Su

**Affiliations:** Key Laboratory for Feed Biotechnology of the Ministry of Agriculture, Feed Research Institute, Chinese Academy of Agricultural Sciences, Beijing 100081, China; xiaolu4444@126.com (X.W.); baiyingguo@caas.cn (Y.B.); huanghuoqing@caas.cn (H.H.); tutao@caas.cn (T.T.); wangyuan08@caas.cn (Y.W.); Wangyaru@caas.cn (Y.W.); luohuiying@caas.cn (H.L.)

**Keywords:** laccase, mycotoxins, aflatoxin B_1_, zearalenone, mediator, detoxification

## Abstract

Aflatoxin B_1_ (AFB_1_) and zearalenone (ZEN) exert deleterious effects to human and animal health. In this study, the ability of a CotA laccase from *Bacillus subtilis* (*Bs*CotA) to degrade these two mycotoxins was first investigated. Among the nine structurally defined chemical compounds, methyl syringate was the most efficient mediator assisting *Bs*CotA to degrade AFB_1_ (98.0%) and ZEN (100.0%). *Bs*CotA could also use plant extracts, including the *Epimedium brevicornu*, *Cucumis sativus* L., *Lavandula angustifolia*, and *Schizonepeta tenuifolia* extracts to degrade AFB_1_ and ZEN. Using hydra and BLYES as indicators, it was demonstrated that the degraded products of AFB_1_ and ZEN using the laccase/mediator systems were detoxified. Finally, a laccase of fungal origin was also able to degrade AFB_1_ and ZEN in the presence of the discovered mediators. The findings shed light on the possibility of using laccases and a mediator, particularly a natural plant-derived complex mediator, to simultaneously degrade AFB_1_ and ZEN contaminants in food and feed.

## 1. Introduction

Mycotoxins are a large group of fungal secondary metabolites that, by contaminating food and feed, are threatening the health of the humans and animals worldwide. Up to date, more than 500 kinds of mycotoxins with much differing structures have been identified [1]. Aflatoxin B_1_ (AFB_1_) and zearalenone (ZEN) are among the most economically important mycotoxins due to their high frequency of contamination in cereals [2]. Being produced by the Aspergilli species including *Aspergillus flavus*, *Aspergillus nomius*, and *Aspergillus parasiticus* [3], aflatoxins cause an economical loss of $1.68 billion in the United States annually [4]. There are more than 20 types of aflatoxins including AFB_1_, AFB_2_, AFG_1_, and AFG_2_, among which AFB_1_ is the most toxic, causing severe liver damage [5]. Zearalenone (ZEN), a mycotoxin with estrogenic potency [6] causing swelling of the vulva and infertility in swine [7], is instead produced by the *Fusarium* species, such as *Fusarium graminearum* and *Fusarium culmorum* [8]. A high concentration of ZEN is found to often accumulate in agricultural commodities, such as *Fusarium*-infected maize and wheat grains, thus similarly bringing a safety risk in food and feed [9,10].

Microbes isolated from soil, nuts, and other environments have been reported to be able to degrade mycotoxins [11,12]. However, most, if not all, discovered enzymes are reported to degrade only a certain type of mycotoxins. In general, among the enzymes capable of degrading mycotoxins, oxidoreductase, including the lignin-oxidizing oxidoreducase, has a wide substrate specificity. It would be, therefore, intriguing to see if these enzymes have activity on multiple mycotoxins.

In contrast to the well-observed wide substrate promiscuity, laccase, an enzyme first identified from the Japanese lacquer tree [13] and later discovered widely distributed in fungi and bacteria [14], is occasionally reported to degrade only one mycotoxin, i.e., either AFB_1_ or ZEN [15,16]. Laccase catalyzes the oxidation of a wide range of phenolic compounds to phenoxy radicals using oxygen as the electron acceptor [17]. Due to its low redox potential, it cannot oxidize chemicals with high redox potentials (such as non-phenolic lignin components) [18]. Its catalytic ability can, however, be enhanced by mediators which are small molecules and substrates of laccase [19]. By reacting with a laccase, mediators form free radicals that can further react with substrates. Artificial synthetic chemicals such as 2,2′-Azino-bis(3-ethylbenzothiazoline-6-sulfonic acid) (ABTS) and 1-hydroxybenzotriazole (HBT) and natural phenolic compounds, such as vanillin and ferulic acid, can all act as mediators and expand the substrate scope of a laccase [20]. The laccase-mediator system has been frequently used for pulp bio-bleaching [21] and dye decolorization [22] but rarely studied in mycotoxins detoxification [15]. Recently, the Ery4 laccase from *Pleurotus eryngii* was reported to degrade AFB_1_, FB_1_ (fumonisin B_1_), OTA (ochratoxin A), ZEN, and T-2 toxin in the presence of certain mediators [23]. However, it is not known if the ability to degrade mycotoxins is a common feature shared by laccases and if other structurally defined chemicals and even natural plant extracts could also act as a mediator.

Due to the wide substrate specificity of laccases, it is expected that they may degrade multiple mycotoxins rather than only one. It is also expected that the degrading activity may not be limited to one particular laccase but should instead be a common feature shared by laccases of different origins. Moreover, the presence of an appropriate mediator, regardless of being chemically synthesized or naturally derived, would have the potential to improve the degrading ability of a laccase.

## 2. Results

### 2.1. Biochemical Properties of the CotA laccase from *Bacillus subtilis* (BsCotA)

**Figure 1:**
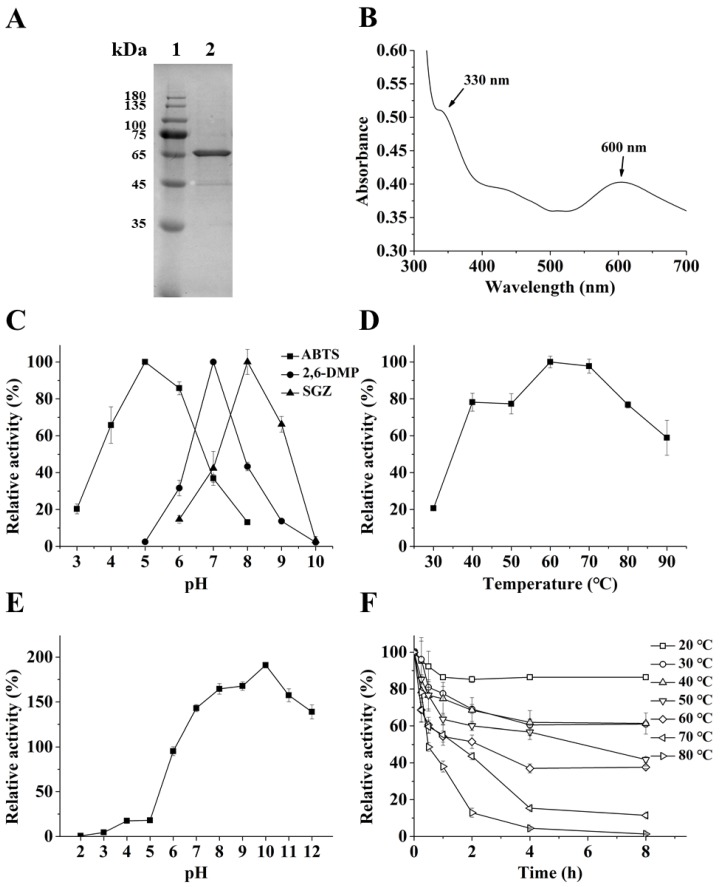
Biochemical properties of recombinant CotA laccase from *Bacillus subtilis* (*Bs*CotA). (**A**) Purification of the recombinant *Bs*CotA. Lane 1, Marker; 2, *Bs*CotA. (**B**) UV–visible spectrum of *Bs*CotA. (**C**) Effect of pH on *Bs*CotA activity. The substrates used were 2,2′-Azino-bis(3-ethylbenzothiazoline-6-sulfonic acid) (ABTS), 2,6-dimethoxy phenol (2,6-DMP), and syringaldazine (SGZ). (**D**) Effect of temperature on *Bs*CotA activity. (**E**) pH Stability. (**F**) Thermostability. For (**D–F**), the activity was assayed using ABTS as the substrate. Each assay was carried out with three independent biological replicates.

The *B. subtilis* cotA gene (1542 bp) encodes a laccase of 513 amino acids with no predicted signal peptide. The recombinant *Bs*CotA was successfully expressed as an intracellular soluble form in *E. coli* BL21 (DE3) harboring pET-28a-CotA induced with isopropyl-β-D-thiogalactoside (IPTG) in the presence of CuSO_4_. The recombinant protein was purified through immobilized metal affinity chromatography. The purified *Bs*CotA was ~65 kDa (Figure 1A) on the SDS-PAGE gel, slightly larger than the calculated 63.8 kDa. The UV–visible spectrum of the purified enzyme (Figure 1B) showed two peaks at 600 and 330 nm, corresponding to the typical T1 copper (600 nm) center and the T3 binuclear copper center (330 nm) of a laccase, respectively [24].

As expected, *Bs*CotA displayed activity toward ABTS, DMP, and syringaldazine (SGZ) (Table 1), the canonical substrates of laccase with structures listed in Table 2 [25,26,27] with the K_m_ being 178.73, 1.35, and 118.80 μM, and k_cat_ being 7.72, 2.73, and 2.39 s^−1^.

Using ABTS as the substrate, the specific activity of purified *Bs*CotA was 6.7 U/mg. *Bs*CotA was stable in the presence of Cu^2+^, and Co^2+^ even increased the enzyme activity to 202.8% (Figure S1). However, Al^3+^, Mn^2+^, and Cr^3+^ reduced the enzyme activity by 39.6, 25.0, and 31.9%, respectively. *Bs*CotA displayed different pH optimums of 5.0, 7.0, and 8.0 for ABTS, DMP, and SGZ, respectively (Figure 1C). The optimal temperature of *Bs*CotA was 60 °C, and it had high activity from 40 to 90 °C. The enzyme had about 60% activity at 90 °C (Figure 1D). *Bs*CotA was unstable under acidic condition. Its residual activity at pH 6.0 was 95.3%, and dramatically decreased to 18.0% at pH 5.0 and only 0.6% at pH 2.0. However, there was no loss of enzyme activity (but rather increments of residual activity up to 1.9-fold at pH 10.0) at the pH above 7.0 after 1 h of incubation, indicating high stability of *Bs*CotA at alkaline conditions (Figure 1E). *Bs*CotA is a thermostable enzyme—it showed 55.2 and 38.0% activity after 1 h of incubation at 70 and 80 °C, respectively (Figure 1F). The character of high pH stability and thermostability is similar to previously reported *Bs*CotA laccases from *B. subtilis* [28] and other *Bacillus* species [29].

### 2.2. Degradation of Mycotoxins by BsCotA with Structurally Defined Chemical Compounds as Mediators

**Figure 2:**
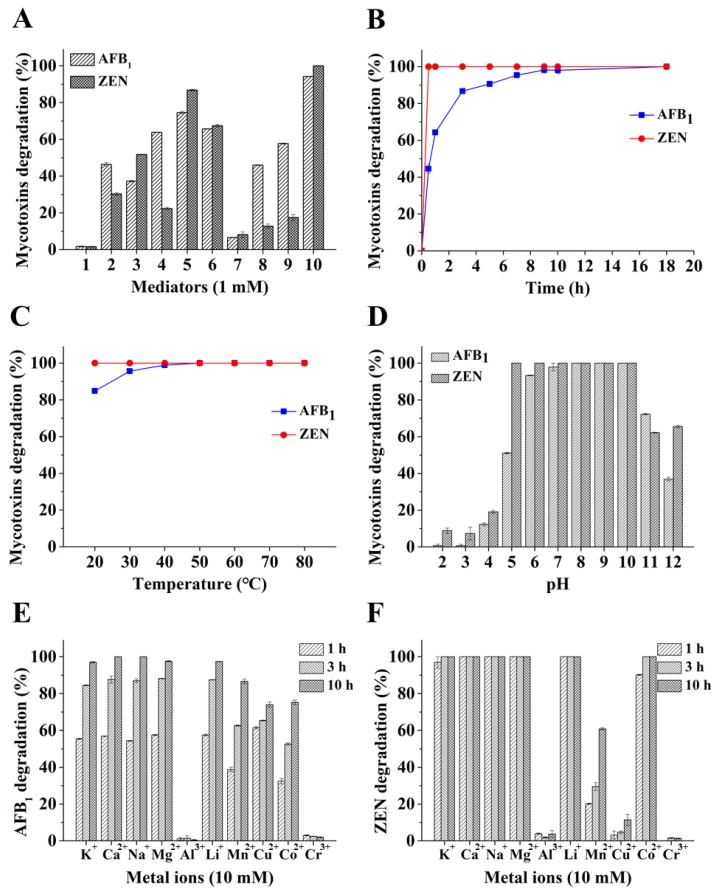
Screening structurally defined chemical compounds as a mediator of *Bs*CotA in the degradation of Aflatoxin B_1_ (AFB_1_) and zearalenone (ZEN). (**A**) Identification of structurally defined chemical compounds as effective mediators. AFB_1_ and ZEN (5 μg/mL each) were individually incubated with *Bs*CotA (0.03 U/mL) and one of the mediators (1 mM) in 50 mM Tris–HCl buffer (pH 7.0) at 30 °C for 10 h. The compounds used were: 1, no compound control; 2, *p*-coumaric acid; 3, syringic acid; 4, vanillin; 5, syringaldehyde; 6, caffeic acid; 7, 1-hydroxybenzotriazole (HBT); 8. gallic acid; 9, vanillic acid; 10, methyl syringate. (**B**) Time-course analysis of AFB_1_ and ZEN transformation by *Bs*CotA in the presence of methyl syringate. AFB_1_ and ZEN (5 μg/mL each) were individually incubated with *Bs*CotA (0.03 U/mL) and 1 mM of methyl syringate in 50 mM Tris–HCl buffer (pH 7.0) at 30 °C for 10 h. The samples were periodically taken for HPLC analysis. Effects of (**C**) temperature and (**D**) pH on AFB_1_ and ZEN transformation by *Bs*CotA in the presence of methyl syringate. For (**C**), AFB_1_ and ZEN (5 μg/mL each) were individually incubated with *Bs*CotA (0.03 U/mL) and 1 mM of methyl syringate in 50 mM Tris–HCl buffer (pH 7.0) at a temperature ranging from 20 to 80 °C for 10 h. For (**D**), the incubation was carried out in a buffer with the pH ranging from 2.0 to 12.0. Impacts of (**E**) metal ions on AFB_1_ and (**F**) ZEN transformation. AFB_1_ and ZEN (5 μg/mL each) were individually incubated with *Bs*CotA (0.03 U/mL) and 1 mM of methyl syringate in 50 mM Tris–HCl buffer (pH 7.0) and one of the metal ions (10 mM) at 30 °C for 10 h. Each assay was carried out with three independent biological replicates.

A few laccases have been reported with modest ability to degrade one of the two mycotoxins AFB_1_ and ZEN [16,30]. However, only negligible activity was observed for *Bs*CotA under the tested condition (Figure 2A). It is well-known that, by utilizing a mediator, laccase can react with more recalcitrant compounds with higher redox potentials. Therefore, chemical compounds with a defined chemical structure were first screened for a possible mediator role, which may assist in degrading AFB_1_ and ZEN (Table 2). Addition of the chemicals including methyl syringate, caffeic acid, syringaldehyde, and vanillin dramatically promoted the degradation of the two mycotoxins by *Bs*CotA, as determined by monitoring the disappearance of AFB_1_ and ZEN. In this screening, the highest degradation rate was obtained with methyl syringate (94.2% for AFB_1_ and 100% for ZEN) in a 10 h reaction. The second highest rate was achieved for syringaldehyde, with degradation efficiencies of 74.6 and 86.9% for AFB_1_ and ZEN, respectively (Figure 2A). The least efficient mediator was HBT, with degradation rates of 6.6 and 8.2% for AFB_1_ and ZEN, respectively. However, even in the presence of methyl syringate, there was no degradation for DON, OTA, and FB_1_ (data not shown).

In the presence of methyl syringate, a time-course analysis indicated that the AFB_1_ degradation rate was 44.5% after 30 min, increased steadily to 90.8% at 5 h, and then gradually ascended to 98.0% after 10 h. The degradation of ZEN was faster than AFB_1_, with a rate of 100% after 30 min (Figure 2B).

Mycotoxins are thermostable molecules [31]. AFB_1_ can withstand a temperature as high as 160 °C [32] while ZEN is only decomposed by 3.2% after heating at 100 °C for 15 min [33]. The AFB_1_ degradation rate rose from 84.8 to 100% when the reaction temperature changed from 20 to 50 °C. However, at the tested conditions, the ZEN degradation rates remained as 100% from 20 to 80 °C (Figure 2C). AFB_1_ could be degraded almost completely at the pHs ranging from 6.0 to 10.0 and for ZEN, this pH range was extended to 5.0 to 10.0. When the pHs were lower (<6.0 for AFB_1_ and <5.0 for ZEN) or above 10.0, the degradation rates were inhibited: 12.3 and 19.1% at pH 4.0, 72.3 and 62.2% at pH 11.0 for AFB_1_ and ZEN, respectively (Figure 2D). The metal ions K^+^, Ca^2+^, Na^+^, Mg^2+^, and Li^+^ had no obvious effect on AFB_1_ and ZEN degradation since the two mycotoxins were nearly completely degraded after 10 h (Figure 2E). In contrast, in the presence of Mn^2+^, Cu^2+^, Co^2+^, Cr^3+^, and Al^3+^, AFB_1_ degradation rates were 86.6, 74.0, 75.2, 2.0, and 0.39% after 10 h of incubation, respectively (Figure 2E). With these five metal ions, the ZEN degradation rates were 60.8, 11.4, 100, 1.26, and 3.7%, respectively (Figure 2F). The decrease of mycotoxin degradation rates in Cr^3+^ and Al^3+^ was clear to a larger extent than reduction of the activity of *Bs*CotA on ABTS in the presence of these metals.

### 2.3. Degradation of Mycotoxins by BsCotA with Plant Extracts as a Natural Mediator

**Figure 3:**
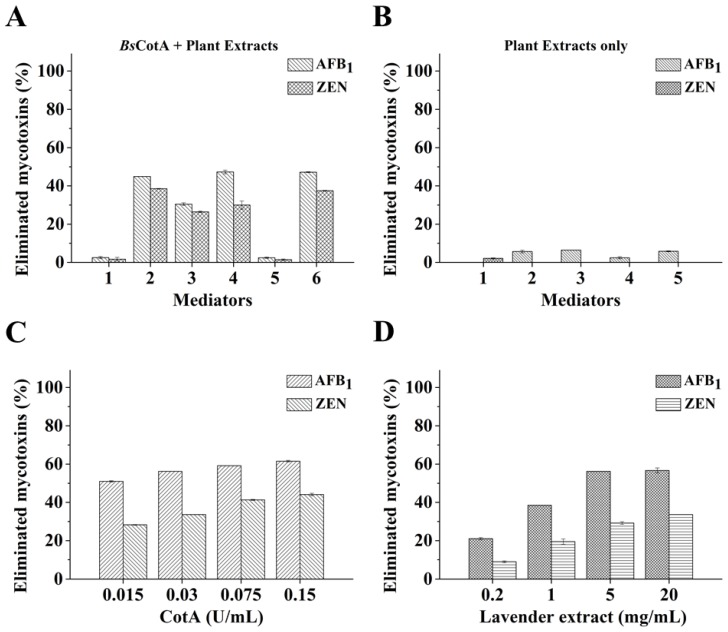
AFB_1_ and ZEN degradation by *Bs*CotA using plant extracts as a mediator. (**A**) Screening of plant extracts as a mediator of *Bs*CotA in AFB_1_ and ZEN degradation. AFB_1_ and ZEN (5 μg/mL each) were individually incubated with *Bs*CotA (0.03 U/mL) and one of the plant extracts (5 mg/mL) in 50 mM Tris–HCl buffer (pH 7.0) at 30 °C for 10 h. 1, no mediator control; 2, *Epimedium brevicornu* extract; 3, *Cucumis sativus* L. extract; 4, *Lavandula angustifolia* extract; 5, *Asparagus officinalis* extract; 6, *Schizonepeta tenuifolia* extract. (**B**) AFB_1_ and ZEN incubated only with plant extracts. AFB_1_ and ZEN (5 μg/mL each) were individually incubated only with one of the plant extracts (5 mg/mL) in 50 mM Tris–HCl buffer (pH 7.0) at 30 °C for 10 h. 1, *E. brevicornu* extract; 2, *C. sativus* L. extract; 3, *L. angustifolia* extract; 4, *A. officinalis* extract; 5, *S. tenuifolia* extract. Effects of (**C**) *Bs*CotA and (**D**) *L. angustifolia* extract concentrations on the transformation rates of AFB_1_ and ZEN. For (**C)**, AFB_1_ and ZEN (5 μg/mL each) were individually incubated with varying concentrations of *Bs*CotA (0.015, 0.03, 0.075, and 0.15 U/mL) and *L. angustifolia* extract (5 mg/mL) in 50 mM Tris–HCl buffer (pH 7.0) at 30 °C for 10 h. For (**D**), the two mycotoxins (5 μg/mL each) were individually incubated with *Bs*CotA (0.03 U/mL) and *L. angustifolia* extract (0.2, 1, 5, and 20 mg/mL) at 30 °C for 10 h. Each assay was carried out with three independent biological replicates.

Although methyl syringate was an efficient mediator assisting degradation of AFB_1_ and ZEN by *Bs*CotA, whether plant extracts (complex mixtures of natural chemical compounds) can serve as an efficient mediator is not known. As a derivative of plants they may be potentially preferred by the food and feed industries. Therefore, we chose several plant extracts to test their efficacy in aiding *Bs*CotA to degrade AFB_1_ and ZEN. Among the tested plant extracts, the *Epimedium brevicornu* (epimedium), *Lavandula angustifolia* (lavender), and *Schizonepeta tenuifolia* (schizonepeta) extracts showed similar efficiency by having the degradation rates of 44.9, 47.3, and 47.2% for AFB_1_ and 38.5, 30.0, and 37.4% for ZEN, respectively (Figure 3A). Not all tested plant extracts is an effective mediator for *Bs*CotA-mediated mycotoxins degradation. In the absence of *Bs*CotA, only a modest part of mycotoxins (particularly AFB_1_) was observed to disappear in HPLC analysis (Figure 3B).

Using *L. angustifolia* extract as a representative laccase mediator, it was demonstrated that the mycotoxins transformation rates were proportional to the concentration of *Bs*CotA and *L. angustifolia* extract. With 5 mg/mL of *L. angustifolia* extract, the degradation rates of AFB_1_ and ZEN gradually ascended from 51.0 to 61.5% (for AFB_1_) or from 28.2 to 44.1% (for ZEN) when the concentration of *Bs*CotA increased from 0.015 to 0.15 U/mL (Figure 3C). With the same amount of *Bs*CotA (0.03 U/mL), the degradation rates of AFB_1_ and ZEN increased from 21.0 to 56.7% for AFB_1_ or from 8.9 to 33.6% for ZEN when the *L. angustifolia* extract was added from 0.2 to 20 mg/mL (Figure 3D).

### 2.4. Evidence for AFB_1_ and ZEN Detoxification by BsCotA/Mediator Treatment

**Figure 4:**
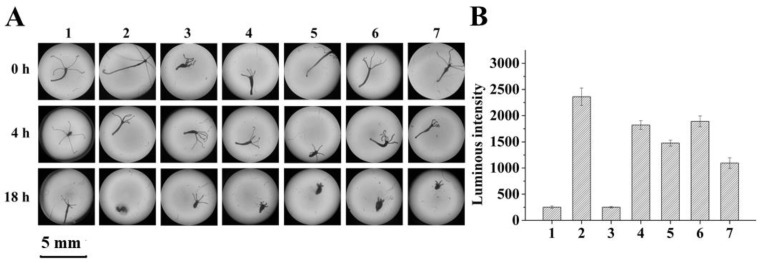
*Bs*CotA-treated AFB_1_ and ZEN led to detoxification as determined by using hydra and BLYES yeast as two model systems. (**A**) Effects of *Bs*CotA treatment on the toxicity of AFB_1_ to hydra. AFB_1_ (5 μg/mL) was treated with one of the *Bs*CotA (0.03 U/mL) mediator systems in 50 mM Tris–HCl buffer (pH 7.0) at 30 °C for 10 h. 1, no AFB_1_ control; 2, untreated AFB_1_; 3–7, AFB_1_ treated with one representative structurally defined chemical mediator methyl syringate (3) and four plant extracts with mediating activity, i.e., *E. brevicornu* extract (4), *C. sativus* L. extract (5), *L. angustifolia* extract (6), and *S. tenuifolia* extract (7). (**B**) Effects of *Bs*CotA treatment on the estrogenic activity of ZEN to the BLYES yeast. ZEN (5 μg/mL) was treated with one of the *Bs*CotA (0.03 U/mL) mediator systems in 50 mM Tris–HCl buffer (pH 7.0) at 30 °C for 10 h. 1, no ZEN control; 2, untreated ZEN; 3–7, ZEN treated with methyl syringate (3), *E. brevicornu* extract (4), *C. sativus* L. extract (5), *L. angustifolia* extract (6), and *S. tenuifolia* extract (7). Each assay was carried out with three independent biological replicates.

To determine if mycotoxins degradation may lead to detoxification, two simplified systems were employed for AFB_1_ and ZEN, respectively, which allows a first glimpse of the role of laccase/mediator treatment. For AFB_1_, growth of hydra was inspected in a culture containing AFB_1_ treated or non-treated with one of the *Bs*CotA/mediators. These mediators, including one structurally defined chemical (methyl syringate) and four plant extracts (*E. brevicornu* extract, *Cucumis sativus* L. (cucumber) extract, *L. angustifolia* extract, and *S. tenuifolia* extract) were selected due to their high mediating efficiencies. The hydra finally collapsed at 18 h when incubated with non-treated AFB_1_ (Figure 4A). In contrast, the hydra remained alive when they were incubated with AFB_1_-treated with *Bs*CotA in the presence of methyl syringate, *E. brevicornu* extract, *C. sativus* L. extract, *L. angustifolia* extract, or *S. tenuifolia* extract (Figure 4A).

ZEN exhibits its toxicity mainly by exerting an estrogenic activity. An engineered *Saccharomyces cerevisiae* strain BLYES has an intracellular estrogen receptor which upon the binding of ZEN will promote emission of bioluminescence [34]. Therefore, BLYES was grown to an optical density of 0.6 and incubated for 6 h with ZEN treated with or without one of the *Bs*CotA mediators. The non-treated ZEN was carefully diluted to a concentration where the most sensitive detection of ZEN degradation could be achieved (data not shown). With methyl syringate, *E. brevicornu* extract, *C. sativus* L. extract, *L. angustifolia* extract, and *S. tenuifolia* extract as mediators, the *Bs*CotA treatment significantly mitigated the estrogenicity of ZEN as manifested by the significantly lower bioluminescenc (*p* < 0.01) compared to the BLYES strain with ZEN alone (Figure 4B). We concluded that, with these simplified model systems, the *Bs*CotA-mediator systems could at least partially detoxify the AFB_1_ and ZEN mycotoxins.

### 2.5. Degradation of AFB_1_ and ZEN by a Fungal Laccase

**Figure 5:**
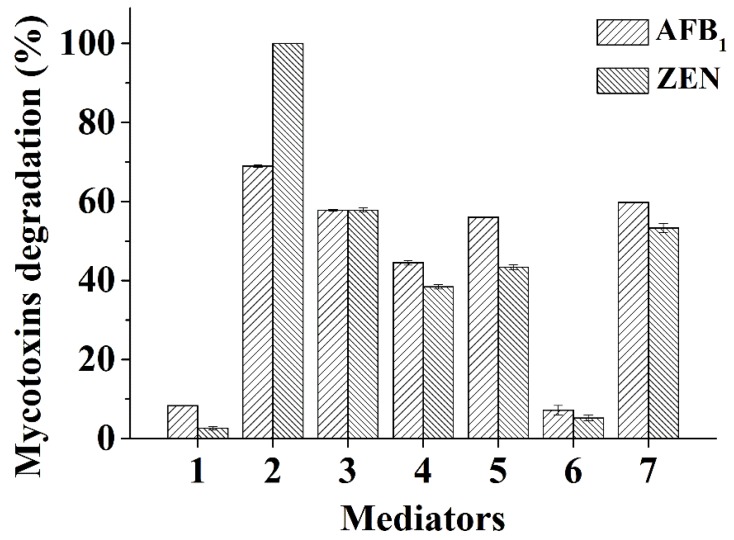
Degradation of AFB_1_ and ZEN by the *Ganoderma* sp. laccase in the presence of a certain mediator. AFB_1_ and ZEN (5 μg/mL each) were individually incubated with a *Ganoderma* sp. laccase (0.03 U/mL) and 1 mM of methyl syringate or one of the plant extracts (5 mg/mL) at 30 °C for 10 h. 1, no mediator control; 2, methyl syringate; 3, *E. brevicornu* extract; 4, *C. sativus* L. extract; 5, *L. angustifolia* extract; 6, *A. officinalis* extract; 7, *S. tenuifolia* extract. Each assay was carried out with three independent biological replicates.

To determine if the identified mediators can also be used for other laccases, a publicly available *Ganoderma* laccase was tested for AFB_1_ and ZEN transformation using the structurally defined chemicals and natural mediators including methyl syringate (1 mM) or one of the five plant extracts (*E. brevicornu*, *C. sativus* L., *L. angustifolia*, *Asparagus officinalis* (asparagus), and *S. tenuifolia* extracts, at 5 mg/mL). The highest degradation rates for AFB_1_ and ZEN were obtained with methyl syringate (69.0% and 100%, respectively) (Figure 5). Except the *A. officinalis* extract, all other plant extracts achieved transformation rates of 44.5~59.8% and 38.4~57.8% for AFB_1_ and ZEN, respectively.

## 3. Discussion

Laccase is widely distributed in fungi, plants, bacteria, and insects [35]. *Bs*CotA, the *B. subtilis* spore coat laccase, is responsible for the biosynthesis of the brown spore pigment [26]. The specific activity of recombinant *Bs*CotA (6.7 U/mg) was similar to the laccase from *Klebsiella pneumoniae* (7.1 U/mg) [36], but lower than those of *Bacillus pumilus* MK001 (73 U/mg) [37] and *B. pumilus* DSM 27 (200 U/mg) [38]. ABTS, DMP, and SGZ are all substrates of *Bs*CotA, a feature shared by *Bs*CotA and other laccases [39]. The wide substrate specificity is related to a flexible lid-like region close to the substrate-binding site mediating substrate accessibility [40]. Apparently, the pH optimums of *Bs*CotA were largely different, with 5.0 for ABTS, 7.0 for DMP, and 8.0 for SGZ, respectively. The shift of pH optimum against another substrate has also been observed for the *Bacillus licheniformi*s LS04 laccase (pH 4.2, 6.6, and 6.2 for ABTS, DMP, and SGZ, respectively) [29] and the *Thermus* sp. 2.9 laccase (pH 5.0 and 6.0 for ABTS and DMP, respectively) [41]. One prominent feature of the *Bs*CotA laccase is its thermo-tolerance and resistance to alkaline pH. Although it lost 95.6% of its activity after 1 h of incubation at pH 3.0, its activity increased significantly after *Bs*CotA was incubated at pH ranging from 7.0 to 12.0 [29,42]. Alkaline pH-stimulated activity has been reported for several laccases; however, the reason remains unknown [29,42,43].

Unlike the many bacterial laccases that are produced in an insoluble form [24,44], the *Bs*CotA laccase gene was successfully expressed in *E. coli* in a soluble and active form. In combination with the merits of high thermostability and pH stabillity, *Bs*CotA appears to be an ideal model for studying laccase-mediated mycotoxins degradation. Our initial assessment of five major mycotoxins (AFB_1_, ZEN, DON, OTA, and FB_1_) revealed that only AFB_1_ and ZEN, but not the other three mycotoxins, can be degraded by *Bs*CotA (data not shown). However, even for AFB_1_ and ZEN, the degradation rates were as low as 1.7 and 1.6%, respectively. This is similar to the *P. eryngii* laccase Ery4, which has no noticeable activity on mycotoxins in the absence of a mediator [23], but in contrast to the *Trametes versicolor* laccase, which can directly act on AFB_1_ [15]. Therefore, the character of a laccase significantly impacts on its direct degradation of mycotoxins, with the underlying mechanism awaiting further elucidation.

Mediators can expand the scope of a laccase and the laccase-mediator system (LMS) has been widely used to degrade organics [45,46]. Nevertheless, despite a previous achievement [23], much remains unknown about the use of a mediator (especially natural mediators) in combination with a laccase to degrade mycotoxins. The current analyses indicated that a range of compounds, either structurally defined or as naturally occurring complex mixtures, could act as mediators to assist AFB_1_ and ZEN degradation. Some structurally defined chemicals, including syringaldehyde, *p*-coumaric acid, and HBT have been reported to mediate AFB_1_ and ZEN degradation by the Ery4 laccase from *P. eryngii*, albeit with much lower efficiency for AFB_1_ [23]. One reason for the observed discrepancy in degradation efficiencies could be from the different pH values of the reaction systems, i.e., pH 7.0 in our study (Figure 2D) and pH 5.0 in the previous investigation. However, the specific combination of laccase/mediator may also be one factor affecting the degradation efficiency. For example, our findings identified novel structurally defined chemical mediators in addition to reported ones, with methyl syringate having strikingly high efficiency to mediate AFB_1_ and ZEN degradation by *Bs*CotA when compared with the published mediators (Figure 2A). Moreover, the *Ganoderma* sp. laccase/methyl syringate was less efficient in AFB_1_ degradation (compare Figure 2A and Figure 5), although the redox potential of a fungal laccase is commonly known to be higher than that of a bacterial laccase. This is also suggestive of the importance of specific combinations of a laccase/mediator system. Again, the *P. eryngii* Ery4 laccase/mediator can degrade FB_1_ and OTA [23], which was nevertheless not observed in our study for both the bacterial (*Bs*CotA) and fungal (*Ganoderma* sp.) laccases (data not shown). This phenomenon merits further investigation. In addition, while our study clearly indicated that both bacterial and fungal laccases can be used in mycotoxins degradation, the discrepancy in the ability and efficiency of mycotoxin degradation points to the necessity of screening various laccases (and their combination with a mediator) to obtain one with desirable degrading properties.

Most of the mediators tested in this study have a phenolic structure, while HBT is a =N–OH type of mediator [47]. However, they all use a hydrogen-atom transfer (HAT) mechanism to mediate degradation [48]. Note that, although the degradation rates of AFB_1_ and ZEN was similar in the presence of part of the tested mediators (*p*-coumaric, syringic acid, syringaldehyde, caffeic acid, HBT, and methyl syringate), the rates were much different in the presence of rest of them (vanillin, gallic acid, and vanillic acid). Syringic acid/syringaldehyde/methyl syringate and vanillin/vanillic acid share similar structures. Therefore, it is possible that there are structural determinants associated with the differentiated degradation rates. Comparing the structures of syringic acid, syringaldehyde, and methyl syringate suggested that the existence of a methyl group could facilitate electron abstraction from the mediator [18]. Analyzing the laccase catalyzed degradation products of AFB_1_ and ZEN in the presence of methyl synringate as a representative mediator is under way.

Plant extracts consist of a wealth of naturally synthesized phenol or aniline derivative compounds [20]. With the success in screening of structurally defined chemical mediators, it is hypothesized that some plant extracts may also have the ability to mediate degradation of AFB_1_ and ZEN. Four out of five tested plant extracts turned out to be indeed effective in mediating AFB_1_ and ZEN degradation. Among the four plants, *C. sativus* L. is edible while the other three have been widely used in Chinese traditional medicine [49,50,51]. Therefore, the combination of a laccase/natural compound combination has potential in treating AFB_1_- and ZEN-contaminated food and feed. Note, it has to be pointed out that the current evaluation of toxicity of degradation products used hydra and yeast systems, which are much simplified and cannot represent the true scenarios where mycotoxins exert their roles in vivo. Therefore, it undoubtedly requires systematic determination of the toxicity of degradation products using the mammalian cells and even animals as models for the realization of using laccase/mediator systems in the detoxification of AFB_1_ and ZEN. It is also expected that the diversity and complexity of plant extracts provide a possibility to identify a plant extract(s) (or possibly, one or more of their component chemicals) of high activity in mediating AFB_1_ and ZEN degradation without generating toxic products.

Note, compared to methyl syringate, the rates of degradation were low for all four plant extracts even when the concentrations of the laccase and plant extracts were increased. It is expected that, by screening more plant extracts, highly efficient natural mediators may be obtained. In addition, two laccases have been reported to synergize in decolorization of indigo carmine [52] and mediators can also synergize in assisting the removal of pentachlorophenol by a laccase [53]. Herein, we have demonstrated that a fungal laccase can also be used to degrade AFB_1_ and ZEN. While this discovery proved our hypothesis that degradation of mycotoxins is applicable to laccases of both bacterial and fungal origins, with the knowledge of synergy among laccases and mediators, by carefully selecting the laccase and mediator components, it is possible that the efficiency of using plant extracts can be much improved. Since laccase is classified in a large while still expanding CAZy family (Auxiliary Activity Family 1, http://www.cazy.org/AA1.html), the current finding will help discovery of new, robust enzymes in mycotoxin degradation. In facing the worldwide challenge of controlling mycotoxin contamination, the present study offers an opportunity of using a limited number of enzymes such as laccase to simultaneously degrade multiple and much differing mycotoxins, specifically AFB_1_ and ZEN in this investigation.

## 4. Conclusions

In summary, the *B. subtilis* CotA laccase was heterologously expressed in *E. coli* in a biologically active form. *Bs*CotA possesses high thermostability and resistance to alkaline pH. Using methyl syringate as a mediator, *Bs*CotA is very efficient in AFB_1_ and ZEN degradation. In addition, four out of five tested natural plant extracts could also be used as a mediator of *Bs*CotA for the degradation of these two mycotoxins, albeit with a lower efficiency. A commercially available laccase of fungal origin could also degrade the two mycotoxins. Our study hints the potential of using the laccase-mediator system, particularly with plant extracts as a mediator, to simultaneously degrade AFB_1_ and ZEN contaminants in food and feed.

## 5. Materials and Methods

### 5.1. Chemicals and Other Materials

Aflatoxin B_1_ (AFB_1_), zearalenone (ZEN), deoxynivalenol (DON), 2,2’-azino-bis(3-ethylbenzothiazoline-6-sulphonic acid (ABTS), 2,6-dimethoxy phenol (DMP), syringaldazine (SGZ), and methyl syringate were purchased from Sigma-Aldrich (St. Louis, MO, USA). FB_1_ (fumonisin B_1_) and OTA (ocharatoxin A) were purchased from Pribolab (Beijing, China). DNA polymerase, T4 ligase, acetonitrile, and trifluoroacetic acid were purchased from Thermo Fisher Scientific (Waltham, MA, USA). Vanillin, *p*-coumaric, syringic acid, syringaldehyde, caffeic acid, 1-hydroxybenzotriazole (HBT), gallic acid, isopropyl-β-D-thiogalactoside (IPTG), and kanamycin were purchased from Solarbio (Beijing, China). Ni-NTA agarose was purchased from QIAGEN (Hilden, Germany). The fungal laccase from *Ganoderma* sp. was purchased from Sunson (Yinchuan, Ningxia, China). Plant extracts from *E. brevicornu*, *C. sativus* L., *L. angustifolia*, *A. officinalis*, and *S. tenuifolia* were purchased from Ciyuan Biotech (Xi’an, Shanxi, China). All other chemicals were of analytical grade or chromatographically pure, and were commercially available.

### 5.2. Bacterial Strains and Culture Conditions

*Bacillus subtilis* 168 was maintained on Luria-Bertani (LB) slants at 4 °C in our lab. The *Escherichia coli* XL10 was used for gene cloning and plasmid propagation. The *E. coli* BL21 (DE3) strain was used for expression of the laccase. The *B. subtilis* and *E. coli* strains were cultivated at 37 °C with constant shaking at 220 rpm in Luria-Bertani (LB) broth.

### 5.3. Cloning, Expression, and Purification of Recombinant BsCotA

The cotA gene was amplified by polymerase chain reaction (PCR) using the primer pair of *Bs*CotA-F (5′-GCTGAATTCACACTTGAAAAATTTGTGGATGC-3′) and *Bs*CotA-R (5′-GCTGCGGCCGCTTTATGGGGATCAGTTATATC-3’) from the genomic DNA of *B. subtilis*. The amplified product was gel purified and restriction digested with *Eco*RI and *Not*I and ligated into the pre-digested pET-28a(+) vector to obtain pET-28a-CotA. The recombinant plasmid was transformed into *E. coli* BL21 (DE3) competent cells for gene expression. The *E. coli* BL21(DE3) strains harboring pET-28a-CotA were cultured in LB medium supplemented with 50 μg/mL of kanamycin at 37 °C overnight with shaking at 220 rpm. The pre-culture was then inoculated into 200 mL fresh LB medium and the culture was continued at 37 °C for approximately 2 h. When the optical density at 600 nm (OD_600_) reached 0.6–0.8, the cells were induced with 0.5 mM IPTG and 2 mM CuSO_4_. The temperature was changed to 16 °C and the culture was continued for 15 h for induction of *Bs*CotA expression. Cells were harvested by centrifugation (12, 000 × *g*, 10 min, 4 °C) and the pellets was re-suspended in a binding buffer (50 mM Na_2_HPO_4_–NaH_2_PO_4_ buffer, 500 mM NaCl and 20 mM imidazole, pH 7.5). Cells were disrupted by ultrasonication, followed by centrifugation (12,000 × *g*, 20 min, 4 °C) to remove cell debris. The soluble extract was passed through a Ni-affinity column resin, then the resin was washed with 50 mM Na_2_HPO_4_–NaH_2_PO_4_ buffer (pH 7.5) containing 500 mM NaCl and 20 mM imidazole to remove the non-specifically bound proteins. The bound *Bs*CotA was eluted with 50 mM Na_2_HPO_4_–NaH_2_PO_4_ buffer (pH 7.5) containing 500 mM NaCl and 40/60/80/100/200/300/500 mM imidazole. The eluted fractions containing pure *Bs*CotA were pooled and changed to a protein storage buffer (50 mM Tris–HCl buffer, 150 mM NaCl, pH 7.5) by ultrafiltration.

### 5.4. Determining the Laccase Activity

The laccase activity was measured by monitoring the oxidation of 1 mM ABTS (ε_420_ = 36,000 M^−1^·cm^−1^) in a 50 mM sodium acetate buffer (pH 4.8). The absorbance was measured at 420 nm for 3 min by incubating the samples at 30 °C. One unit (1 U) of laccase activity was defined as the amount of enzyme that produced 1 μmol of product per minute under the standard assay conditions.

### 5.5. Biochemical Characterization of Recombinant BsCotA

The purified *Bs*CotA in 50 mM Tris–HCl buffer (pH 7.5) was first scanned for absorbance at 300–700 nm with a UV–vis spectrometer (Biotek Winooski, VT, USA) at room temperature. One mM each of ABTS, DMP (ε_470_ = 49, 600 M^−1^·cm^−1^), and SGZ (ε_530_ = 65,000 M^−1^·cm^−1^) were used in determining the optimal pH of *Bs*CotA at 30 °C. The buffers used were 100 mM Na_2_HPO_4_–citric acid (pH 3.0 to 7.0), 100 mM Tris-HCl buffer (pH 7.0 to 9.0), and 100 mM glycine–NaOH buffer (pH 9.0 to 10.0). The pH stability of *Bs*CotA was determined at 20 °C by incubating the appropriately diluted enzyme in buffers from pH 2.0 to 12.0 for 1 h. The residual activity was measured using ABTS as the substrate. The optimal temperature of *Bs*CotA was examined at its optimal pH (for ABTS) at varying temperatures ranging from 30 to 90 °C. The thermostability of *Bs*CotA was investigated at temperatures ranging from 20 °C to 80 °C by incubating the appropriately diluted enzyme in 100 mM Na_2_HPO_4_–citric acid buffer (pH 6.0) for various time periods. The samples were collected at 0.25, 0.5, 1, 2, 4, and 8 h and the residual activities were measured.

The effect of various metal ions (Al^3+^, Mn^2+^, Cu^2+^, Co^2+^, Cr^3+^) on the laccase activity of *Bs*CotA was measured by adding 1 mM each of the metal ions to the reaction system. The kinetic parameters of purified *Bs*CotA were determined at its optimal pH at 30 °C using different concentrations of ABTS (0.05–4 mM, pH 5.0), DMP (0.1–16 mM, pH 7.0), or SGZ (0.05–1 mM, pH 8.0), respectively. The kinetic parameters were calculated by non-linear regression fitting of the data to the Michaelis–Menten equation using GraphPad Prism 5 (Version 5; GraphPad Prism, La Jolla, CA, USA, 2016).

### 5.6. Mycotoxins Degradation by the Laccase/Mediator Systems

To prepare plant extracts, grinded plant powders were mixed with water, shaken at 150 rpm for 1 h, and then centrifuged at 12,000 × *g* for 5 min at 4 °C to remove the debris. *Bs*CotA (0.03 U/mL against ABTS, the same amount applicable to the *Ganoderma* sp. laccase) was incubated with each of the five major mycotoxins (AFB_1_ and ZEN, 5 μg/mL; DON and FB_1_, 10 μg/mL; OTA, 50 μg/mL) in 50 mM Tris–HCl buffer (pH 7.0) supplemented with one of the structurally defined chemicals (*p*-coumarate, syringic acid, vanillin, syringaldehyde, caffeic acid, HBT, gallic acid, methyl syringate, or vanillic acid, 1 mM for each) or plant extracts from *E. brevicornu*, *C. sativus* L., *L. angustifolia*, *A. officinalis*, or *S. tenuifolia* (5 mg/mL each). Each reaction was repeated three times.

For *Bs*CotA-methyl syringate degradation of AFB_1_ and ZEN, a time-course analysis was carried out at 30 °C by periodically taking out the samples, in which three volumes of DMSO were added to terminate the reaction. To investigate the effect of pH on AFB_1_ and ZEN transformation by *Bs*CotA, the reaction was performed for 10 h in different pH buffers using methyl syringate as the mediator. The buffers were 100 mM glycine–HCl buffer (pH 2.0), 100 mM Na_2_HPO_4_–citric acid buffer (pH 3.0 to 7.0), 100 mM Tris–HCl buffer (pH 7.0 to 9.0), and 100 mM glycine–NaOH buffer (pH 9.0 to 12.0), respectively. To investigate the effect of temperature on AFB_1_ and ZEN transformation in the *Bs*CotA-mediator (methyl syringate) system, the reactions were performed for 10 h at different temperatures ranging from 20 to 80 °C. The effect of metal ions (K^+^, Ca^2+^, Na^+^, Mg^2+^, Al^3+^, Li^+^, Mn^2+^, Cu^2+^, Co^2+^, and Cr^3+^) on the mycotoxins transformation was also studied. The metal ions (10 mM each) were individually added to the reaction system with *Bs*CotA–methyl syringate. The effect of enzyme and mediator (*L. angustifolia* extract) concentrations on AFB_1_ and ZEN transformation were also studied by varying the *Bs*CotA concentration from 0.015 to 0.15 U/mL and the *L. angustifolia* extract from 0.2 to 20 mg/mL. Each reaction was repeated three times.

### 5.7. Hydra Assay

The hydras were maintained clean and free from bacteria and fungi contamination by treating them with dilute iodine solution (2.7 μg/mL). The assay was performed by exposing the hydra to AFB_1_ treated or non-treated with *Bs*CotA and one of the mediators. Fifty μg/mL of AFB_1_ were incubated with a *Bs*CotA (0.03 U/mL) mediator system in 50 mM Tris–HCl buffer (pH 7.0). The reaction was carried out at 30 °C for 10 h. Each test dish contained 1 mL of test solution and three normal healthy hydra. The hydras were observed under a Carl Zeiss Axio Vert.A1. microscope (Jena, Germany). The hydras were examined for signs of toxicity at 0, 4, and 18 h, respectively. The toxic endpoint was determined by the “tulip” or “disintegration” stage of the hydra [54].

### 5.8. BLYES Assay

The *S. cerevisiae* BLYES strain was inoculated into 30 mL yeast minimal medium ((NH_4_)_2_SO_4_) 1.7 g/L, CuSO_4_ 12 mg/L, FeSO_4_ 684 µg/L, KH_2_PO_4_ 11.6 g/L, KOH 3.6 g/L, MgSO_4_ 171 µg/L, D-(+)-glucose 20 g/L, biotin 20 µg/L, pantothenic acid 400 µg/L, inositol 1 mg/L, pyridoxine 400 µg/L, thiamine 400 µg/L, adenine 42.7 mg/L, arginine HCl 17.1 mg/L, aspartic acid 100 mg/L, glutamic acid 85.5 mg/L, histidine 42.73 mg/L, isoleucine 25.64 mg/L, lysine HCl 25.64 mg/L, methionine 17.1 mg/L, phenylalanine 21.4 mg/L, serine 320.4 mg/L, threonine 192 mg/L, tyrosine 25.7 mg/L) in a baked 250 mL glass flask. The cells were cultured at 28 °C with constant shaking at 200 rpm to an OD_600_ of 0.6. ZEN (5 μg/mL) was treated with one of the *Bs*CotA-mediator systems. Appropriately diluted reaction products were mixed with 200 µL BLYES and the estrogenicity was checked by measuring the bioluminescence of the cells collected 4 to 8 h post treatment [34]. Each reaction was repeated three times.

### 5.9. HPLC Analysis

Degradation of AFB_1_ and ZEN was analyzed through HPLC using a SHIMADZU 20A series instrument (Shimadzu, Kyoto, Japan) with an Agilent ZORBAX SB-C18 column (5 μm, 4.6 mm × 250 mm) (Agilent, Santa Clara, CA, USA). The elution condition for AFB_1_ and ZEN was set as no acetonitrile (ACN), 4 min; 0–100% ACN, 25 min; 100% ACN, 6 min, at a flow rate of 0.8 mL/min. AFB_1_ and ZEN were monitored at 365 nm or 316 nm [55,56], respectively. The degradation percentage was calculated using the following formula: mycotoxin degradation (%) = (S_control_ − S_sample_/S_control_) × 100%, where S_control_ and S_sample_ were the peak areas of the mycotoxins in the control and sample, respectively [30].

### 5.10. Statistical Analyses

The software SPSS 17.0 developed by IBM (Version 17.0; IBM, Armonk, NY, USA, 2008) was used for statistical analysis of the data.

## Figures and Tables

**Table 1:** Kinetic parameters of *Bs*CotA.

Substrate	*k_cat_* (s^−1^)	*K_m_* (μM)	*k_cat_/K_m_* (M^−1^·s^−1^)
ABTS	7.72 ± 0.67	178.73 ± 49.53	(4.56 ± 0.90) × 10^4^
DMP	2.73 ± 0.18	(1.35 ± 0.27) × 10^3^	(2.09 ± 0.31) × 10^3^
SGZ	2.39 ± 0.08	118.80 ± 12.36	(2.04 ± 0.25) × 10^4^

**Table 2:** Model substrates for the laccase and structurally defined chemicals as potential mediators used in this study.

Class	Compound	Structure
**Laccase Model Substrate**		
	ABTS	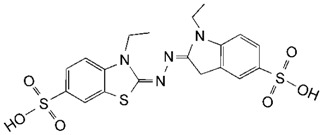
	DMP	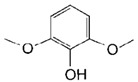
	SGZ	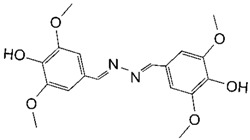
**Structurally Defined Chemicals as Potential Mediators**		
	*p*-Coumaric acid	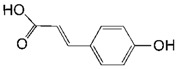
	Syringic acid	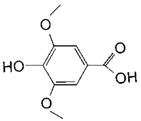
	Vanillin	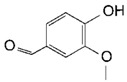
	Syringaldehyde	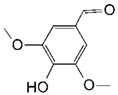
	Caffeic acid	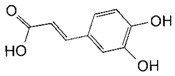
	1-Hydroxybenzotriazole	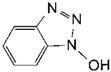
	Gallic acid	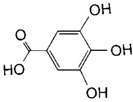
	Vanillic acid	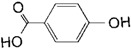
	Methyl syringate	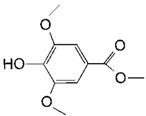

## Supplementary Materials

Supplementary materials can be found at https://www.mdpi.com/2072-6651/11/10/609/s1. Figure S1: Activity of BsCotA in the presence of metal ions.

## Abbreviations

AFB_1_	Aflatoxin B_1_
ZEN	Zearalenone
DON	Deoxynivalenol
FB_1_	Fumonisin B_1_
