# Supplementary material for: Degradation of Aflatoxin B1 and Zearalenone by Bacterial and Fungal Laccases in Presence of Structurally Defined Chemicals and Complex Natural Mediators
Source: Toxins (Basel). 2019 Oct 22;11(10):609. doi: 10.3390/toxins11100609 (PMC6832423; doi:10.3390/toxins11100609)
Supplement: Supplementary file 1 [file toxins-11-00609-s001.pdf]

Supplementary Materials: Degradation of Aflatoxin B1 and Zearalenone by Bacterial and Fungal Laccases in Presence of Structurally Defined Chemicals and Complex Natural Mediators

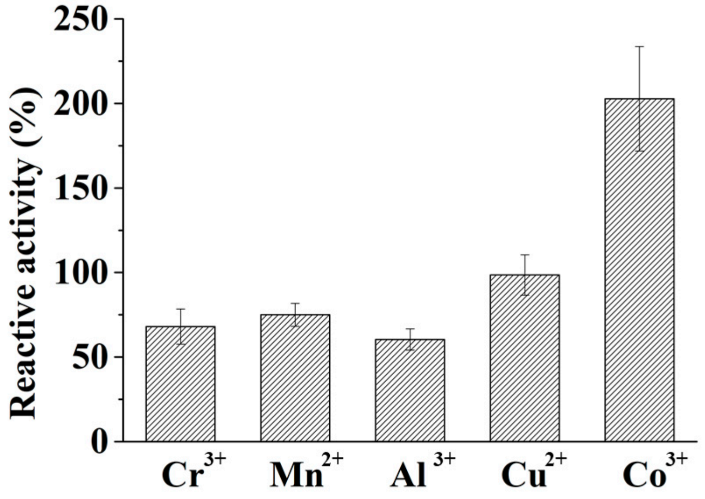

Figure S1. Activity of BsCotA in the presence of metal ions.
